# Growing ganja permission: a real gate-way for Thailand’s promising industrial crop?

**DOI:** 10.1186/s42238-022-00121-4

**Published:** 2022-03-06

**Authors:** Sarana Rose Sommano, Tibet Tangpao, Tanachai Pankasemsuk, Voranate Ponpanumas, Yuthana Phimolsiripol, Pornchai Rachtanapun, Shashanka K. Prasad

**Affiliations:** 1 Department of Plant and Soil Sciences, Faculty of Agriculture, Chiang Mai University, Chiang Mai, Thailand; 2 Cluster of Agro Bio-Circular-Green Industry (Agro BCG), Chiang Mai University, Chiang Mai, Thailand; 3 Department of Animal and Aquatic Science, Faculty of Agriculture, Chiang Mai University, Chiang Mai, Thailand; 4 School of Agro-Industry, Faculty of Agro-Industry, Chiang Mai University, Chiang Mai, Thailand; 5 Department of Biotechnology and Bioinformatics, Faculty of Life Sciences, JSS Academy of Higher Education and Research, Mysuru, India

**Keywords:** Cannabis, Ganja, Legalization, Marijuana, Thai sticks, Thailand

## Abstract

The current revision to Thailand’s Narcotics Act (B.E. 2563) permits Thai corporations to produce cannabis (ganja) for therapeutic purposes, as well as conduct beneficial research and development in science and agriculture. While ganja possession, distribution, and use are still illegal in Thailand, the law removes certain elements of *Cannabis sativa* (including hemp) from the narcotic lists as of December 2020 and Thailand's narcotics board plans to remove them totally from the lists before the last quarter of 2022. The Thai Food and Drug Administration (Thai FDA) board maintains the exclusive licensing authority to assess applications and provide authorization due to the complexity of the registration process. In this view, we analyzed the guidelines for obtaining cannabis production license, and it was apparent that the announced law was in-line with regulations set-out by many countries in terms of security and prevention of misuse. The other criteria however fall merely onto the government gains, rather than public interests. To avoid the claimed state monopoly, several types of licensing should be issued in the future, depending on the genuine purpose of the farmers. The complete regulation process and conditions for obtaining a ganja growing license in Thailand are highlighted and discussed in this review.

## Background

**Fig. 1:**
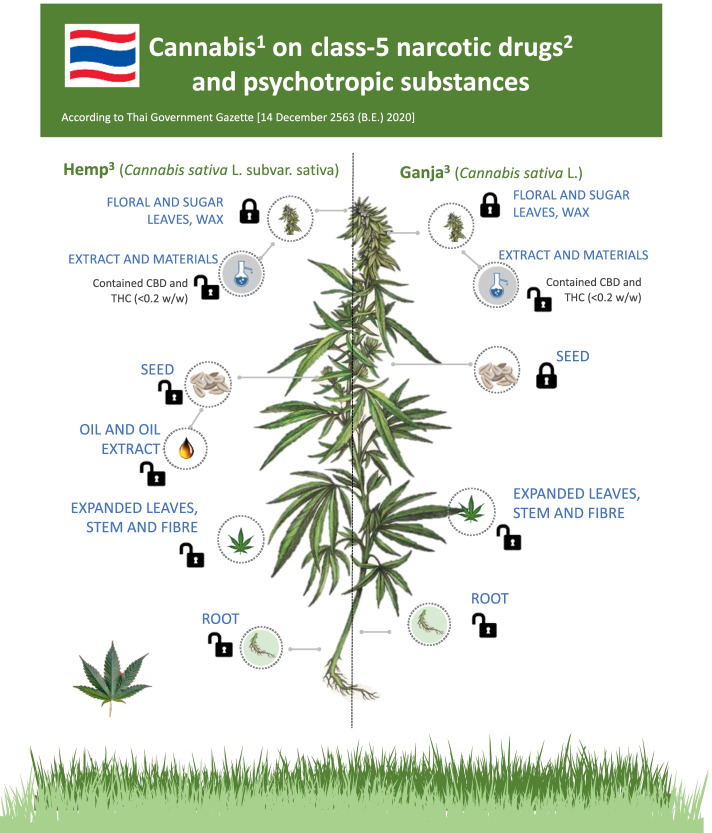
Ganja on class-5 narcotic drugs and psychotropic substances of the Thai government gazette effective December, 2020. Superscript digit one indicates the following: under approved permission by Thai FDA. Superscript digit two indicates the following: the Thai government listed ganja, kratom, opium, and hallucinogenic mushrooms in class-5 of narcotics classification in the Narcotics Act (Saingam et al. 2013). The removal of ganja off the lists is being examined for amendment of the Act in 2022, but it must be published in the official Royal Gazette before then (Reuters 2022). Superscript digit three indicates the following: only produced in Thailand

Apart from kratom (*Mitragyna speciosa* Korth) and a tablet methamphetamine or yaba, ganja or marijuana (*Cannabis sativa* L.) has remained the most commonly reported type of illicit drugs used in Thailand for the last 20 years (Angkurawaranon et al. 2018). In traditional alternative medicine, these plant-based medications are known for their functional properties, and Thailand is well known for having an ideal climate for production (Chouvy 2019; Tipparat et al. 2012). More importantly, since the late 1960s, Thailand has been noted for developing the unique technique for ganja growing that produces thick inflorescences dense to the stem known as “Thai sticks” (Chouvy 2019; Kravanja 2016). Nowadays, it is commonly agreed that the cannabis either hemp or ganja is from the same species of *C. sativa* L., and therefore, growing these plants had been prohibited in Thailand since 1979. Since 2007, however, attempts have been made to legalize hemp for textile purposes (Tipparat et al. 2014; Sommano et al. 2020). In 2018, Thai legislation formally legalized ganja as a class-5 narcotic drug and psychotropic substance for therapeutic purposes, although recreational use of the substance is still prohibited (Cannacata 2020; Kanato et al. 2020). The current permission intends to make ganja one of Thailand's future industrial crops, with benefits for research, agriculture, tourism, and the local market, as well as protecting Thai verities’ intellectual property (Cannacata 2020). As from December 2020, certain parts of ganja and hemp (subsp. sativa) as well as their extracts and by-products from extracting process with the content of tetrahydrocannabinol (THC) no greater than 0.2% were removed from the narcotic list (Gazette and Health Mo 2020). The permission allows stem, root, leaves with no bud or florescence, hemp seed and hemp seed oil, under granted permission, to be used freely (Fig. 1). The plant parts and substances other than these are still considered drugs of psychotropic potential under the provisions of the Thai Narcotics Act (B.E. 2522) (1979). For distribution of cannabis-related drugs, the penalty is a maximum of 15 years in jail and a fine of up to 1.5 million THB, with lesser penalties for manufacturing, importation, or exportation depending on the quantity (Leechaianan and Longmire 2013; Aroonsrimorakot et al. 2019). Meanwhile, charges for cannabis-related drug usage carry a maximum sentence of 1 year in prison and a maximum fine of 20,000 THB. Nonetheless, after the legal revisions took effect in 2018, research revealed a massive increase in the number of ganja users in Thailand (Kanato et al. 2020). This number may rise in the future as the public at large becomes more aware of scientific evidence and the therapeutic effects of cannabis in the treatment of illnesses (Ratwichit and Jitkuakul 2019; Thaikla et al. 2018). The new rule allows licensed traditional medicine professionals and modern medical practitioners to dispense licensed medicinal grade cannabis products and Thai Traditional Medicine formulations (Zinboonyahgoon et al. 2020).

Although Thailand was the first country in Asia to amend its narcotics-control laws in an attempt to promote the use of cannabis for medical purposes (Zinboonyahgoon et al. 2021; Jialin et al. 2020), experts have opined that the amendment is very limited, allowing only licensed research and development with public institutes and consumption with a medical certificate, raising the question of whether it will or will not benefit the nations and, more importantly, Thai farmers (Beatty 2020; Parpart 2020). The purposes of current review are to access the status of legalization and to discuss the advancement and limitation(s) of existing regulation(s) for Thai cannabis growers. The presented information herein will be advantageous for farmers and entrepreneurs, alike, abiding by these new law and regulation.

### Significant of ganja permit regulations for Thai public health

While the public is concerned about the change of Thailand's cannabis law, legalization is exclusively for medicinal purposes or as drug regulation in general (Rägo and Santoso 2008). In 2018, the World Health Organization (WHO) expert committee on drug dependence had agreed on the appropriate level of international controls for cannabidiol (CBD), a phytocannabinoid devoid of psychoactive effects to be used for medical purposes (Mayor 2019); the Thai government has adopted this to the law a year later on February 18, 2019 (Zinboonyahgoon et al. 2020; Rehm et al. 2019). CBD has recently been studied in preclinical and clinical trials and proven to have a potential pharmacotherapy for treating symptoms of a variety of neuropsychiatric illnesses, including addiction, anxiety, psychosis, motility problems, and epilepsy (Premoli et al. 2019). This active ingredient binds to key brain receptors involved in the metabolic and neuroinflammatory pathologies and a variety of brain functions including the type-1 cannabinoid receptor (CB1R) and the type-2 cannabinoid receptor (CB2R) (Kumar et al. 2019). The metabolic syndromes are thought to be linked to a change in systemic inflammation, with CBD playing a major role in the mitigation (Mastinu et al. 2018). Patients who want to use cannabis for medical purposes must first get a prescription from a practitioner, dentist, or a registered Thai-traditional medical professional. The diagnosis must be made based on evidence-based medicinal procedures derived from highly credible research studies, clinical specialists, and the expected outcome of patients (Akasin 2019). The existing regulation’s effects for public health are primarily focused on three types of medical complications (Akasin 2019; Nonthasawadsri 2020; Suphanchaimat and Pavasuthipaisit 2018) as the following:

1. The conditions with highly supportive research evidences including difficult-to treat epilepsy, side effects from chemotherapy (nausea and vomiting), spasticity in multiple sclerosis, and intractable neuropathic pain and HIV
2. The conditions that require further scientific evidences including Parkinson’s disease, Alzheimer’s disease, demyelinating disease, anxiety disorder, and patients in a palliative care and final stage of cancer
3. The conditions that may be privilege from ganja treatment but without adequate research evidences
4. In 1985, the United States Food and Drug Administration (US FDA) approved two synthetic equivalents of THC, Marinol® (dronabinol) and Cesamet® (nabilone), for alleviating nausea and vomiting associated with cancer chemotherapy in patients who have failed to respond to conventional antiemetic therapies (Brunetti et al. 2020). In the decades later, these therapeutic cannabinoid products were used to treat different forms of chronic pains (Croxford 2003) and neurological disorders such as multiple sclerosis (Pertwee 2002), epilepsy (Cortesi and Fusar-Poli 2006), and other movement disorders (Müller-Vahl et al. 1999), following by the approval of other synthetic drugs including Syndros® (dronabinol, oral solution) and Canemes® (nabilone, capsules). In Europe, however, the herbal preparations in the forms of decoctions, oils, and sprays or simply inhaled with a vaporizer (i.e., Volcano® and pen vaporizers) are preferable (Brunetti et al. 2020; Hall 2018). After the release of the current law, Thai FDA has also approved natural cannabis products including cannabis extracts produced mainly by Government Pharmaceutical Organization (GPO), Thai traditional recipes and cannabis oil (DTAM®) manufactured by department of Thai traditional and Alternative Medicine (Nonthasawadsri 2020).

### The position of Thailand regulation within the broader cannabis regulation environment across the world

Cannabis and other substances, including the herbal forms of resin, extracts, and tinctures, were included in Schedule I of the Single Convention on Narcotic Drugs, making their use, possession, production, manufacture, export, import, distribution, and commerce illegal except for medical and research purposes (Hall 2018; UN 1972). Since the early 1990s, there has been an increase in global interest in the legalization of cannabis for therapeutic purposes (Hakkarainen et al. 2015). This movement began in 1996 in California, where 215 initiatives were approved in the favor of medicinal cannabis legalization. Following that, uses of medical marijuana was allowed in 28 states in the USA, with 18 more allowing some forms of recreational marijuana usage (Dyer 2017; Hollenbeck and Uetake 2020). In the Netherlands, possession of small amounts of cannabis imposes no offense and it is among the leading countries with the altered policy to reduce the penalties relating to narcotic drugs (MacCoun and Reuter 1997). However, there are no other European Union countries which officially legalize cannabis supply for recreational purposes, but legislative models are currently purposed along with decriminalization for home cultivation in Belgium, Czech Republic, and Spain (Hughes et al. 2017; Belackova et al. 2019; Bone et al. 2018). Decriminalization for personal cultivated marijuana was also a part of the drug law (de jury) in three Australian states and territories (South Australia, Australian Capital Territory (ACT), and Northern Territory), as well as Jamaica (Belackova et al. 2019). Marijuana producers in Canada must apply for licenses through the health ministry and must pass security checks and quality-control inspections, as per the “Medical Marijuana Access Regulations” (MMAR) of 2001 (Eggertson 2013; Fischer et al. 2015). It was estimated that more than 2% of Canadian citizens relied on medical marijuana treatments and nearly hundreds of individuals had applied to become licensed growers impacting a ten billion dollars business revenue (Hollenbeck and Uetake 2020; Fischer et al. 2015). In 2018, Canadian government has passed the legalization of recreational marijuana for adults under certain regulatory in place (Cox 2018). It is generally accepted that legalizing cannabis for patients has often implemented an ever strict regime, for example, the authorities and medical professionals still request evidence-based recognition of cannabis as an approved treatment (Hakkarainen et al. 2015).

Overall, the ultimate goals of ganja legalization in Thailand are primarily to provide access to this substance to patients who rely on it for therapy, as well as to support research and development. The legalization also allows different classes of federal licensees including cultivation (both of hemp and ganja), processing, and others such as research and analytical services. The government pays less attention to the risks connected with illicit markets, juvenile access restrictions, the construction of an appropriate safety and regulatory environment, and the criminal justice system, that are all necessary. Furthermore, rather than promoting fraudulent political policy, information and ground understanding about the ganja’s use of conduct should be provided to the public. We therefore conclude that unless the legalization of ganja in Thailand is for recreational use, the current regulation is comparable with a 1961 single convention on narcotic drugs.

### Permission and growing ganja in Thailand

For growers, industrial hemp and licensed cultivation of ganja, for the purposes of research and scientific investigation, is legal in many places. Furthermore, growing these plants for personal use is also allowed in some countries (Ratwichit and Jitkuakul 2019; Hakkarainen et al. 2015; Potter et al. 2015; Lenton et al. 2015). As of June 2020, Thailand’s cultivation permission was only valid for *C. sativa* L. used for research and domestic medical purposes, and parameters like cultivation area, growing quantity, security, detailed information, and the applicant’s criminal record had to be provided along with a duly filled application form (ONCB 2020; Puttasrijaru 2019). Only a few types of applicants are allowed, according to the protocol, including (i) the government and (ii) public universities whose mission is to conduct research, education in agriculture, medical services, or narcotics control, and (iii) Thai farmers associated with a registered community enterprise, private university, and professional individual with a valid agreement with the mentioned register public sectors (MOPH 2020). To prolong a permission, applicants must report that they have no prior criminal records involving narcotic drugs (class-5) and that their previous work progress demonstrates that they have had a consistent track record. Any change in cultivation quantity must be reported to the Thai FDA, which is part of the Ministry of Public Health (MOPH), in order to revise the agreement. Nonetheless, if the cultivation location changes, the government will consider updating the application. According to this new regulation, 2793 permissions have been granted with as many as 343 cultivation permits (as of December 2021). A number of the farmer community enterprises are involved in these permits, the majority of which have an agreement with the MOPH as a public sector counterpart (Narcotics Control Division Food And Drug Administration (FDA) 2020). After the law came into effect, it was anticipated that over 200,000 individuals were prescribed medical cannabis, with 90% of those being first-time users (Kanato et al. 2020). This has the potential to secure the medicinal cannabis industry’s status while also providing growers with a window of opportunity. However, the addiction stigma that reflects the negative perception of ganja use in Thai culture, adverse side effect, and complex system of safeguard are the challenges for cannabis industry and the success of implementation of this new law (Zinboonyahgoon et al. 2020; Ritmontree et al. 2019). The government’s license monopoly model is also seen as a barrier to private investment (Kirdphol and Junngam 2020).

### Legal propagation

Sharing the same scientific name as marijuana, hemp is known for its utilizable fiber. Additionally, the term “hemp seed” is recognized when the material is used as a source of seed oil. For ganja, the resinous blend of cannabinoids that localized mainly in the trichomes of floral tissues is used for recreational or as therapeutic drugs (Small 2015; Clarke and Merlin 2016). The international criteria, nonetheless, deems CBD as the major cannabinoid composition in the floral tissue of industrial hemp which is typically about 2% weight by weight (w/w) or less, and THC should be less than 0.3% w/w, although the European Union standard is not over 0.2% w/w (Hu et al. 2019). Ganja, on the other hand, is dominated by THC, which frequently reaches 20% w/w (Chandra et al. 2017). Apart from the differences in the levels of cannabinoid, hemp, and ganja are quite difficult to distinguish by their morphologies (Datwyler and Weiblen 2006). Sawler et al. (Sawler et al. 2015) also stated that genetic distinction, such as employing specific genes linked in THC production, is insufficient to distinguish hemp from ganja. Hemp plants are usually tall, unbranched, and grown for a high ratio of fibrous stem-to-floral material, with a higher number of flowers. Only the seed of ganja is listed as a narcotic in Thailand’s existing legislation (Gazette and Health Mo 2020). However, the basis of separation of the ganja and hemp seeds employed by the Thai government has not been clearly described.

**Fig. 2:**
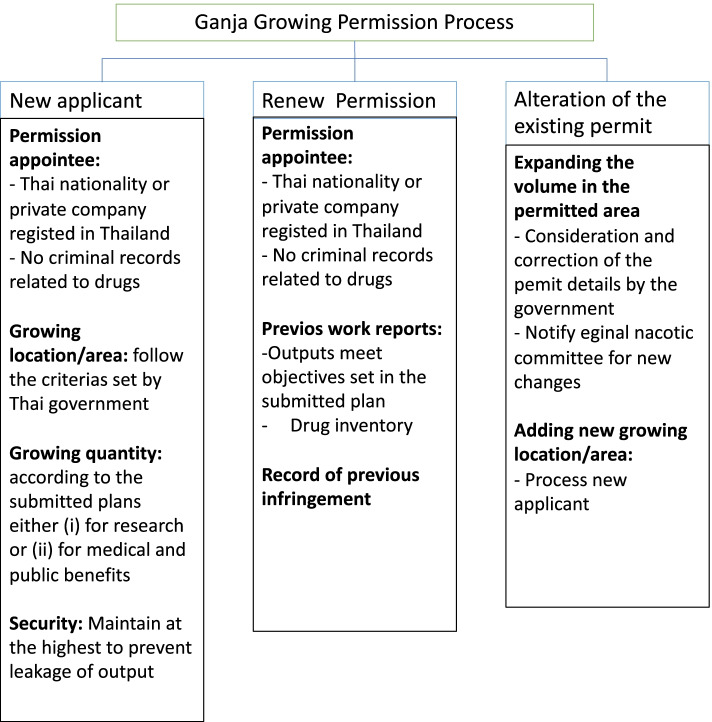
Obtaining ganja growing permit in Thailand (affective June, 2020) (ONCB 2020)

**Fig. 3:**
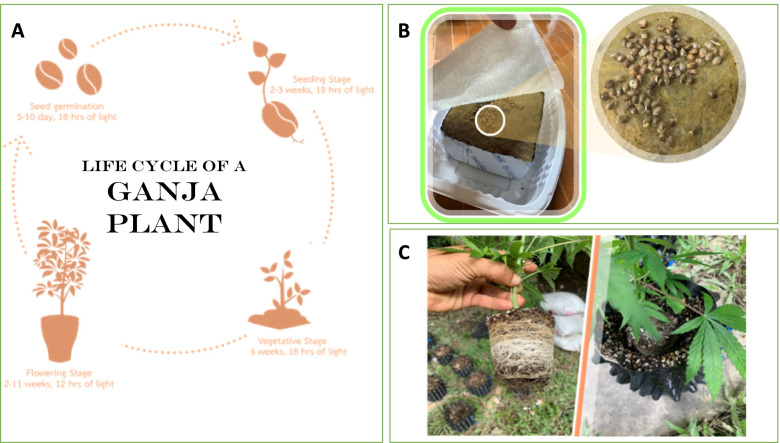
Ganja life cycle (**A**), sprouting cannabis seed (**B**), and vegetative stage of young cannabis plant (**C**) (Mediavilla et al. 1998)

Ganja, like all other angiosperms, has a life cycle that includes seed, seed germination, seedling, vegetative phases, and flowering (Mediavilla et al. 1998) (Fig. 3A). For the pharmaceutical industry, quality control (i.e., the content of medical-grade cannabinoids, biomass, and resin), seedling propagation is considerably less advantageous than vegetative or cutting propagation to avoid male plants and cross pollination of different varieties (Chandra et al. 2020). When sexually propagated, a stable-line germinating seed lot and a standardized growing process are crucial (Chandra et al. 2010). To initiate the rapid growth, sprouting seeds (germinating taproot) require moisture, air, and heat (Fig. 2B). The seed produces its first two leaves, also known as the cotyledons, after 2 weeks. These cotyledons are not true leaves but contain food for the young plant to survive during the first few days. The next set of leaves to appear are the “real leaves,” which resemble the classic cannabis leaves. For the first 3 weeks, the seedling(s) require a minimum of 18 h of light per day to stimulate their growth. Male plants that flower earlier are removed during the early flowering stage, and female plants are kept under the 12-h photoperiod until they reach maturity (Chandra et al. 2017; Chandra et al. 2020). In fact, when grown in hot, dry regions, the temperate hemp seedlings can transform into the narcotic cultivars (Small 2015; Bouquet 1950). Based on the current Thai law, the term ganja seed is ambiguous. However, the legal procedure requires that the applicant for growing license provides the details of seed origins with or without variety name and reports the amount of possession (ONCB 2020). Mother plants of the desired genotypes are cultivated vegetatively with an artificial 18-h artificial light supply for the asexual approach. The vegetative plants are used to produce cutting with rooting hormone, which are maintained in high humidity and continuous light to produce vigorous root system (Potter 2004) (Fig. 3C). The details of additional propagation material, like those required for seed, must be provided throughout the licensing process (ONCB 2020).

### Ganja cultivation requirement

**Fig. 4:**
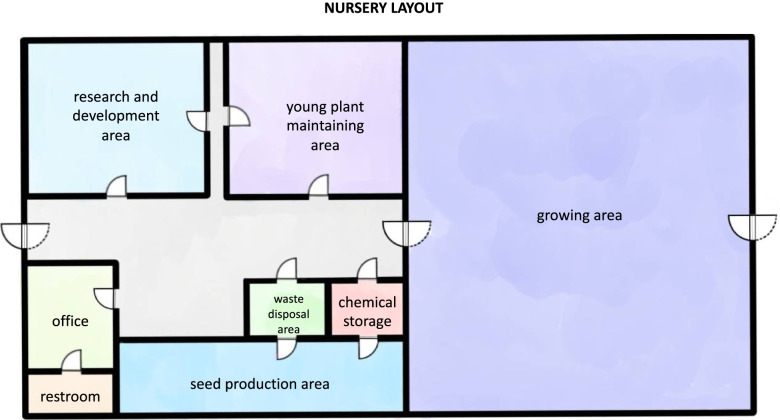
Example of the licensed ganja cultivation nursery layout

The minimal requirements for establishing the cultivation area for medical ganja farmers’ licensing applications in Thailand include the considerations of location, storage and security, controls, and administrative activities for prevention (MOPH 2019). Production, distribution, and utilization plans, as well as the purchasing order or agreement formed between farmers, farm owners, and buyers, should determine the size and amounts of cultivation (ONCB 2020). The initial prerequisite for either indoor or outdoor growing is consent documentation of land or space utilized in a given location (providing evidence of ownership of the property, the geographical address with details of the GPS monitoring system). The growing space has to be protected with secured walls and durable doors with limited number of entrances (including the fire exit). Clear label with the statement “Class-5 narcotic drugs production area” as no less than 3 cm needs to be well exposed. Propagating and cultivation areas are to be separated (Fig. 4). Cultivation must adhere to Good Agricultural Practice (GAP) for herbs and standard operations for cultivating cannabis, with Thai FDA inspections for active cannabinoids and heavy metals on a regular basis (Puttasrijaru 2019; ACFS 2018). Finally, the permission holder has to provide Thai FDA with the standard protocols for logistics control, which includes the harvesting, transportation, and disposal for tracking. Furthermore, full security service (viz. security, electronic access control and CCTV) should be installed with the limit access to the growing site. The requirements for obtaining the permission in Thailand are concluded in Table 1. Similar requirements have been established in Canada for licensed growers. There, they are able to apply for subclasses of licenses, viz. micro, standard or nursery depending on the purpose and scale of production (Application requirements for cannabis cultivation, processing and medical sales licences 2018). In Australia, however, harvesting is excluded from the cultivation license; instead, the grower has to apply for production licenses in addition to growing (ODC 2020).

**Table 1:** The requirements for ganja cultivation permission in Thailand

General requirements	Public and medical purpose	Research purpose
General requirement	The permit only allows growers of the community enterprises or medical professionals with agreements with government institutes and public universities. The written purchasing or distributing order has to be made with potential buyers prior to getting permission.	Government institutes and public universities can directly apply for the permit. However, farmers, medical professionals and private university of Thai nationality of no criminal offenses relating to drug (http://www.criminal.police.go.th/index.php) need to apply together with the public organizations. Growing quantity should agree with the work plan submitted at the permit submission process.
Cultivation site requirement	Address: Complete location address, with GPS tracking location with the valid lease contract for both outdoor and indoor cultivation. Infrastructure: Secure walls with limited access and sign (min 10 × 60 cm) with the statement “สถานที่ผลิตยาเสพติดให้โทษประเภท 5” needs to be presented.	
Security requirement	CCTV needs to be installed around the growing site, entrances, growing and storage areas with Electronic Access Control. Security data must be stored for at least 1 year. Security staffs are required to guard external and internal areas.	
Storage requirement	Output and by-product (waste from harvesting) storage areas are separated with full security system installed.	
Control requirement	The cultivation and harvesting procedures are to follow Good Agricultural Practices (GAP) for herbs. Standard Operation Protocols of ganja cultivation published by Ministry of Public Health are to be followed. The content of active cannabinoids, mycotoxins, and heavy metals are to be randomly examined by Thai FDA in a certified laboratory (ISO/IEC 17025). Cannabis Tracking System and inventory are to be reported to the FDA.	

### Value proposition of the current ganja policy

**Fig. 5:**
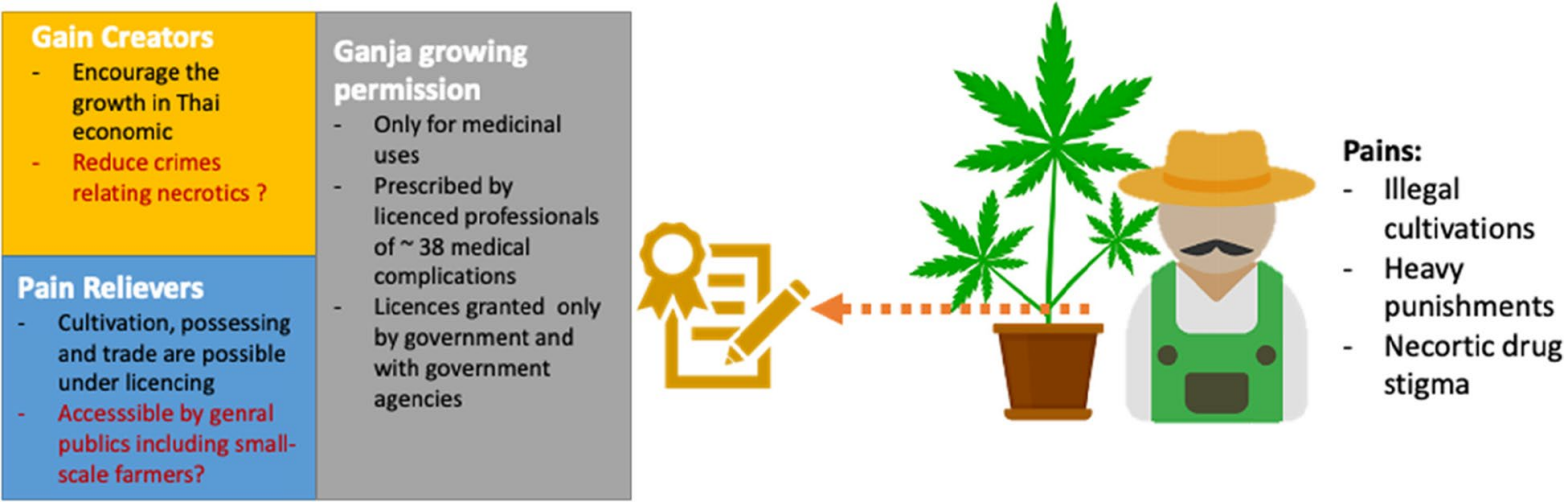
The review of the value propositioning of Thai cannabis permission policy

We used the value proposition canvas to highlight the true value offer to Thai farmers when looking at the cannabis laws (Clark et al. 2012). We looked at the areas of (1) products/services—the national ganja policy—(2) gain creators—how are farmers benefiting from the policy—from the farmers’ perspective—and (3) pain relievers—how do they alleviate the discomfort? (Pokorná et al. 2015). Figure 5 illustrates the value propositioning of the current ganja policy. The ultimate goal of legalizing marijuana cultivation (at least partially) is to protect cannabis-dependent patients' rights to obtain cannabis for medical purposes under the supervision of a licensed physician, as well as to strengthen the country’s pharmaceutical security and prevent pharmaceutical monopolies. (Kanato et al. 2020). According to the Thai FAO, there are around 40 medical conditions for which cannabis can be legally prescribed, and only about 400 medical practitioners who can authorize its usage (Cannacata 2020; Sornpaisarn et al. 2019). Cannabis, on the other hand, was said to have the potential to boost Thailand’s economic growth (Bangkok Post 2019). Therefore, the actual pain from the agricultural sectors was that cultivation, possession, and trade were previously offensive according to the out-date provisions of the Narcotics Act, B.E. 2522 (1979). Furthermore, experts warn that legalizing ganja would cause more harm than good to the country, citing evidence that marijuana can lead to the usage of other narcotics (Saengpassa 2021). While the promulgation of the law was in-line with many countries advancing the uses of cannabis, the Thai growing permission was ambiguous and involving solely by the government approvals (Bone et al. 2018; Kirdphol and Junngam 2020). More crucially, the private sector is not yet explicitly allowed to hold licenses unless they are in cooperative with one of the government's licensees (Cannacata 2020). Overall, it appears that cannabis is not totally legal, and the existing license is unfavorable, particularly for small-scale growers.

Uruguay and Canada are among the first countries to legalize ganja for recreational use. Canada intended to integrate public health and community safety with actual social usage, despite the fact that this was believed to be a tactical response to a powerful illicit market and high crime rates associated to drug sales in Uruguay (Cox 2018). In Thailand, following the implementation of this new law, it was evident that the number of users has increased dramatically. Later in 2022, home cultivation in Thailand may become legalized for non-commercial and medicinal purposes without further licenses (Reuters 2022). As a result, many investors are trying to position themselves within the legal medical market in order to obtain access to the legal recreational market. We believe that the government should invest in cannabis research and development, such as establishing legal age limits, prohibiting driving while intoxicated, and regulating the illicit market.

## Conclusion

The Thai cannabis state’s legalization ambitions have been overwhelming for businesses and individuals looking to benefit from this medical drug from the start, while the current law provided much clearer definition for the narcotic parts from ganja. Because the preconditioning guidelines did not clearly distinguish the production purposes, it was questioned whether the proposed policy was more beneficial to the government than to the public state. The regulation should therefore be amended further to promote ganja as the economic crop of the country and for higher control and prevention of the cannabis misuse.

## Data Availability

Not applicable.

## Abbreviations

CBD: Cannabidiol
GAP: Good Agricultural Practice
GPO: Government Pharmaceutical Organization
MMAR: Medical Marijuana Access Regulations
MOPH: Ministry of Public Health tetrahydrocannabinol
THC: Tetrahydrocannabinol
